# Perioperative blood transfusion does not affect recurrence-free and overall survivals after curative resection for intrahepatic cholangiocarcinoma: a propensity score matching analysis

**DOI:** 10.1186/s12885-017-3745-z

**Published:** 2017-11-14

**Authors:** Pei-Yun Zhou, Zheng Tang, Wei-Ren Liu, Meng-Xin Tian, Lei Jin, Xi-Fei Jiang, Han Wang, Chen-Yang Tao, Zhen-Bin Ding, Yuan-Fei Peng, Shuang-Jian Qiu, Zhi Dai, Jian Zhou, Jia Fan, Ying-Hong Shi

**Affiliations:** 1 Department of Liver Surgery, Liver Cancer Institute, Zhongshan Hospital, Fudan University, 180 FengLin Road, Shanghai, 200032 China; 2 Key Laboratory of Carcinogenesis and Cancer Invasion of Ministry of Education, Shanghai, China; 3 Institutes of Biomedical Sciences, Fudan University, Shanghai, People’s Republic of China

**Keywords:** Intrahepatic cholangiocarcinoma, Hepatectomy, Perioperative blood transfusion, Overall survival, Disease-free survival

## Abstract

**Background:**

Whether perioperative blood transfusions (PBTs) adversely influence oncological outcomes for intrahepatic cholangiocarcinoma (ICC) patients after curative resection remains undetermined.

**Methods:**

Of the 605 patients who underwent curative liver resection for ICC between 2000 and 2012, 93 received PBT. We conducted Cox regression and variable selection logistic regression analyses to identify confounding factors of PBT. Propensity score matching (PSM) and Cox regression analyses were used to compare the overall survival (OS) and disease-free survival (DFS) between the patients with or without PBT.

**Results:**

After exclusion, 93 eligible patients (15.4%) received PBT, compared with 512 (84.6%) who did not receive PBT; the groups were highly biased in terms of the propensity score (PS) analysis (0.096 ± 0.104 vs. 0.479 ± 0.372, *p* < 0.001). PBT was associated with an increased risk of OS (HR: 1.889, 95% CI: 1.446–2.468, *p* < 0.001) and DFS (HR: 1.589, 95% CI: 1.221–2.067, p < 0.001) in the entire cohort. After propensity score matching (PSM), no bias was observed between the groups (PS,0.136 ± 0.117 VS. 0.193 ± 0.167, *p* = 0.785). In the multivariate Cox analysis, PBT was not associated with increased risks of OS (HR: 1.172, 95% CI: 0.756–1.816, *p* = 0.479) and DFS (HR: 0.944, 95% CI: 0.608–1.466, *p* = 0.799). After propensity score adjustment, PBT was still not associated with OS or DFS after ICC curative resection.

**Conclusions:**

The present study found that PBT did not affect DFS and OS after curative resection of ICC.

## Background

Cholangiocarcinoma is the second most prevalent primary liver tumor worldwide, and its 3-year survival rate ranges from 20% to 60% in different regions due to difficulties in its diagnosis and poor responses to current therapies [1–5]. Surgical resection is the only feasible treatment modality that has a curative outcome for patients. Despite fast-paced improvements in surgical technique and experience, there is still a risk of massive blood loss and a subsequent need for blood transfusion. Blood transfusion is a double-edged clinical weapon that maintains blood volume to control hemorrhagic shock, supplies blood components to improve oxygen carrying capacity of blood, and regulates hemostasis by increasing blood coagulation factors. However, transfusions may cause short or severe complications including allergic reactions, hemolytic reactions, and immunosuppression. Several recent studies found PBT may be associated with worse postoperative outcomes for cancer patients [6–8]. However, without random controlled trials, it was debated whether systemic and statistic bias existed that led to this unreliable sign. Indeed, some reports argued that PBT has no impact on tumor recurrence and long-term mortality [9–12]. In this study, we summarize more than a decade of data at our institute and implemented a propensity score matching system to investigate the association between PBT and long-term outcome in ICC patients.

## Methods

### Participants and criteria

**Fig. 1:**
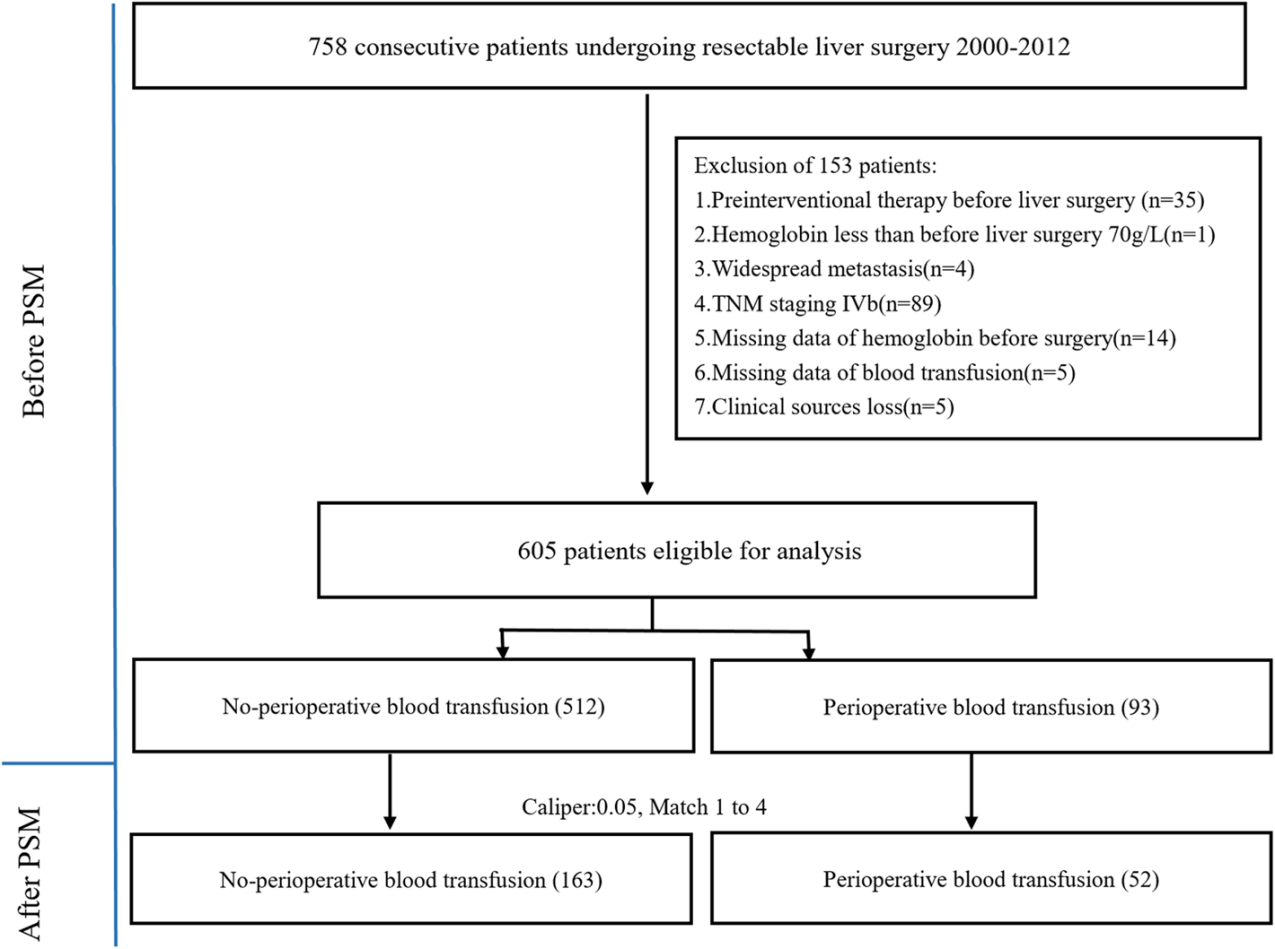
Study flow chart

The study enrolled 758 consecutive ICC patients who underwent curative surgery between 2000 and 2012 at the Liver Cancer Institute, Zhongshan Hospital, Fudan University. All resections were performed or supervised by experienced hepatobiliary surgeons and used standardized procedures [13]. Additionally, all surgical specimens were confirmed by pathologic histology [2]. The following exclusion criteria were used: pre-interventional therapy before liver surgery (*n* = 35, 27 underwent transcatheter arterial chemoembolization (TACE), 1 underwent radiofrequency ablation(RFA), 2 underwent radiotherapy, and 5 underwent RFA plus TACE); hemoglobin less than 70 g/L (*n* = 1); widespread metastasis (*n* = 4); TNM staging IVb (*n* = 89); missing data of hemoglobin before surgery (*n* = 14); missing data of blood transfusion (*n* = 5); and clinical source loss (n = 5) (Fig. 1). The eligible 605 patients included 93 cases who received perioperative allogeneic blood transfusion and 512 cases without transfusion. We defined the perioperative period as the time between the third preoperative day and the seventh postoperative day.

### Data source

All data on the patients’ demographics, morbidity, postoperative mortality, and histological results were obtained from the hospital medical system. The TNM classification was based on the AJCC Cancer Staging Manual, Seventh edition (2010) by springer New York, Inc. All patients were followed-up regularly at outpatient clinics and the Liver Cancer Institute, Zhongshan Hospital, Fudan University. The follow-up results were obtained via telephone by an experienced researcher working in the Liver Cancer Institute. All patients were regularly followed in the outpatient department and tumor markers were measured every 3 months during the first 3 years and thereafter every 6 months until the study end or loss of follow-up. An abdominal ultrasound was performed every 3 months, and abdominal computed tomography or MRI was performed 6 months postoperatively or upon suspected recurrence. The median follow-up time was 20 months (range 0–134 months), and the end follow-up time was November 2015. The primary research endpoint was the death of patient or the end follow-up time, and the secondary endpoint was follow-up dropout. The OS was defined as the period from surgery until death due to any cause. DFS was defined as the duration from surgery until the date of intrahepatic cholangiocarcinoma recurrence. The transfusion of any blood visible components including red blood cells and blood plasma were considered blood transfusion. Blood management, including processing, testing, and transporting, were quality controlled by Shanghai Blood Center. The ABO and Rh status as well as blood cross matching were conducted by the blood department of Zhongshan hospital [14].

### Variables and statistics

The categorical variables are shown as whole numbers and proportions, and the continuous variables are described as the means with standard deviation as appropriate. Two-sided *p* values of <0.05 were considered statistically significant. Statistical analyses were performed using IBM SPSS Statistics 22 and R statistical software. To compare continuous variables that followed Gaussian distributions, t tests were used; the K-Independent-Samples Test (Kruskal Wallis H(K) test) was used for those variables did not follow Gaussian distributions. To compare proportional variables, a Two-Independent-Samples Test (Mann-Whitney U test) was used, and a Two-Related-Samples Test (Wilcoxon Signed Ranks Test) was applied to matched propensity scores [15]; all missing data are reflected in the available data [16]. We examined the following parameters: age, gender, preoperative hemoglobin (Hb), platelets (PLT), aspartate aminotransferase (AST), alanine transaminase (ALT), alpha fetoprotein (AFP), carcinoembryonic antigen (CEA), carbohydrate antigen 19–9 (CA19–9), prothrombin time (PT), international normalized ratio (INR), hepatitis B surface antigen (HBsAg), anti-hepatitis C virus (Anti-HCV), tumor maximum dimension (TMD), tumor node metastasis (TNM), intraoperative blood loss (IBL), degree of differentiation (DD), and transcatheter arterial chemoembolization (TACE). The confounders were measured accurately using univariate Cox regression through an enter variable selection procedure. The full variable selection logistic regression was used to specify a mathematical relationship for the variables related to blood transfusion [17]. The regression models were based on Akaike’s information criterion. We then adjusted for further confounding parameters in the propensity score analysis. This is a useful technique that focuses on the relationship between confounders and the treatment [18, 19]. The “PS MATCHING 3.03” and “SPSS Statistics R Essentials 22.0” and “R-2.15.3-win” R packages [20] were used to perform the matching propensity score analysis. The matching confounders was estimated by the regression models described above, and balance matching showed the values of absolute standard mean difference (SMD). The demographics and characteristics of the matched patients were compared to ensure that there were no significant differences in the baseline settings. We used univariate and multivariate Cox regressions to assess the prognostic value of blood transfusion through balanced data. The GraphPad Prism 6 software was used to draw the survival curves depicting OS and DFS.

## Results

### Demographics and clinical characteristics of the 605 eligible patients before PSM

Of these patients, 93 (15.37%) received blood transfusion, and 512 (84.63%) did not. We described the patient demographics and clinical characteristics for the two groups separately (Table 1). The clinical data for 9 of the 21 variables differed significantly (*P* < 0.05) as a result of a conspicuous bias with pre-described PS (0.096 ± 0.104 vs. 0.479 ± 0.372 *P* < 0.001).

**Table 1:** Demographics and clinical characteristics of 605 eligible patients before PSM

Characteristic	Variable	Before PSM		
		No-PBT	PBT	*P* value
		(*n* = 512)	(*n* = 93)	
Age	Y ± SD	57.42 ± 10.815	56.54 ± 11.801	0.176^a^
Gender	female/ male	190/322	48/45	0.008^c^
Preoperative Hb	g/L ± SD	132.53 ± 16.22	118.63 ± 17.68	0.329^a^
Platelet	1 × 10^9^/L ± SD	179.25 ± 64.02	201.30 ± 97.35	0.038^a^
Total bilirubin	available data	510(99.61%)	92 (98.92%)	
	umol /L ± SD	24.98 ± 55.35	86.79 ± 134.47	<0.001^b^
AST	available data	502(98.05%)	90 (96.77%)	
	U/L ± SD	44.56 ± 87.09	56.21 ± 57.45	<0.001^b^
ALT	available data	509 (99.41%)	92 (98.92%)	
	U/L ± SD	50.40 ± 95.22	57.90 ± 67.28	0.002^b^
AFP	available data	497 (97.07%)	91 (97.85%)	
	ng/mL ± SD	95.28 ± 953.72	93.02 ± 585.95	0.982^b^
CEA	available data	489 (95.51%)	87 (93.55%)	
	ng/mL ± SD	22.42 ± 154.07	61.84 ± 330.14	0.060^b^
CA19–9	available data	486 (94.92%)	86 (92.47%)	
	U/mL ± SD	1152.16 ± 3116.14	2698.09 ± 4923.75	<0.001^b^
PT	available data	506 (98.83%)	91 (97.85%)	
	s ± SD	11.20 ± 9.23	11.87 ± 3.11	0.093^b^
INR	available data	502 (98.05%)	89 (95.70%)	
	Value ±SD	0.98 ± 0.11	0.98 ± 0.14	0.910^b^
HBsAg	available data	506 (98.83%)	93 (100.00%)	
	(−)/(+)	302/204	71/22	0.002^c^
Anti-HCV	available data	502 (98.05%)	93 (100.00%)	
	(−)/(+)	493/9	91/2	0.814^c^
Tumor number	available data	511 (99.80%)	92 (98.92%)	
	1/>1	426/85	81/11	0.259^c^
TMD	available data	509 (99.41%)	93 (100.00%)	
	cm	6.48 ± 3.16	6.92 ± 3.44	0.284^b^
TNM stage	available data	489 (95.51%)	74 (79.57%)	
	I/II/III/IVa	294/80/17/98	47/15/0/12	0.433^c^
Cirrhotic nodule	available data	512 (100.00%)	91 (97.85%)	
	no/ yes	344/168	70/21	0.065^c^
DD	available data	412 (80.47%)	76 (81.72%)	
	I/II-III/IV	2/408/2 (0.49%)	0/76/0 (0.00%)	1.000^c^
IBL	available data	503 (98.24%)	93 (100.00%)	
	<1 L/≥1 L	499/4 (99.20%)	61/32 (65.59%)	<0.001^c^
Preventive TACE	no/yes	474/38 (92.58%)	86/7 (92.47%)	0.972^c^
Propensity Score	available data	501 (84.49%)	92 (15.51%)	
		0.096 ± 0.104	0.479 ± 0.372	<0.001^b^

^a^t test

^b^K-Independent-Samples Test (Kruskal Wallis H(K) test)

^c^Two-Independent-Samples Test (Mann-Whitney U test)

Values are presented as n (%) or mean ± standard deviation(SD)

Hb: hemoglobin; AST: aspartate aminotransferase; ALT: alanine transaminase; AFP: alpha fetoprotein; CEA: carcinoembryonic antigen; CA19–9: carbohydrate antigen 19–9; PT: prothrombin time; INR: international normalized ratio; HBsAg: hepatitis B surface antigen; Anti-HCV: anti-hepatitis C virus; TMD: tumor maximum dimension; TNM: tumor node metastasis; IBL: intraoperative blood loss; DD: The degree of differentiation;

TACE: transcatheter arterial chemoembolization. PSM: propensity score matching; No-PBT: no-perioperative transfusion; PBT: perioperative transfusion

### Confounding factors between the PBT groups and outcome

To investigate whether the existing confounders led to any bias, a univariate Cox regression was conducted to filter out the 8 variables that were associated with the outcome without considering treatment. In the OS Cox regression model, the preoperative Hb, total bilirubin, ALT, CA19–9, anti-HCV, TMD, TNM stage, intraoperative blood loss variables were selected as independent prognostics for ICC patients. In the DFS Cox regression model, CEA, which was an extended prognostic, was picked out. Then, a univariate logistic regression was performed between patients who received blood transfusion and those who did not; the gender, preoperative Hb, PLT, total bilirubin, CA19–9, HBsAg, and intraoperative blood loss differed significantly. After multivariate analysis, only the preoperative Hb, total bilirubin, and intraoperative blood loss were left and were thus considered confounders that had to be adjusted to synthesize all of the regression models (Table 2).

**Table 2:** A univariate Cox regression and a variable selection logistic regression for confounding factors based on Transfusion and No-transfusion

Characteristic	Variables	Before PSM							
		Univariate (OS, COX Regression)		Univariate (DFS, COX Regression)		Univariate (Logistic Regression)		Multivariate (Logistic Regression)	
		HR (95% CI)	*P* value^a^	HR (95% CI)	*P* value^a^	OR (95% CI)	*P* value^a^	OR (95% CI)	*P* value^a^
Age	Y ± SD	1.007 (0.998–1.017)	0.131	1.006 (0.997–1.014)	0.219	0.993 (0.973–1.013)	0.477		
Gender	female	reference	0.121	reference	0.145	reference	0.009	reference	0.506
	male	1.184 (0.956–1.466)		1.163 (0.949–1.425)		0.553 (0.355–0.863)		0.790 (0.393–1.585)	
Preoperative Hb	g/L ± SD	0.988 (0.981–0.994)	<0.001	0.990 (0.985–0.996)	0.001	0.951 (0.937–0.965)	<0.001	0.947 (0.927–0.967)	<0.001
Platelet	1 × 10^9^/L ± SD	1.001 (1.000–1.003)	0.177	1.001 (0.999–1.002)	0.222	1.004 (1.001–1.007)	0.006	1.001 (0.997–1.006)	0.537
Total bilirubin	umol/L + SD	1.003 (1.002–1.004)	<0.001	1.002(1.001–1.003)	0.001	1.007 (1.005–1.010)	<0.001	1.006 (1.003–1.009)	<0.001
AST	U/L ± SD	1.000 (0.999–1.002)	0.487	1.001 (1.000–1.002)	0.205	1.001 (0.999–1.003)	0.24		
ALT	U/L ± SD	1.001 (1.000–1.002)	0.029	1.001 (1.000–1.002)	0.049	1.001 (0.999–1.003)	0.472		
AFP	ng/mL ± SD	1.000 (1.000–1.000)	0.379	1.000 (1.000–1.000)	0.451	1.000 (1.000–1.000)	0.983		
CEA	ng/mL ± SD	1.000 (1.000–1.000)	0.745	1.001 (1.000–1.001)	0.01	1.001 (1.000–1.002)	0.108		
CA19–9	U/mL ± SD	1.000 (1.000–1.000)	<0.001	1.000 (1.000–1.000)	<0.001	1.000 (1.000–1.000)	<0.001	1.000 (1.000–1.000)	0.903
PT	s ± SD	1.000 (0.988–1.012)	0.99	1.008 (0.994–1.022)	0.28	1.007 (0.986–1.028)	0.507		
INR	Value ±SD	2.307 (0.949–5.605)	0.065	1.692 (0.742–3.855)	0.211	0.714 (0.095–5.388)	0.744		
HBsAg	(−)	reference	0.401	reference	0.232	reference	0.003	reference	0.126
	(+)	0.912 (0.737–1.130)		0.882 (0.718–1.084)		0.459 (0.275–0.764)		0.552 (0.258–1.183)	
Anti-HCV	(−)	reference	0.04	reference	0.076	reference	0.814		
	(+)	0.395 (0.163–0.956)		0.507 (0.240–1.073)		1.204 (0.256–5.663)			
Tumor number	1	reference	0.446	reference	0.559	reference	0.261		
	>1	1.122 (0.835–1.507)		1.088 (0.821–1.441)		0.681 (0.348–1.332)			
TMD	cm	1.052 (1.017–1.087)	0.003	1.057 (1.025–1.090)	<0.001	1.041 (0.975–1.113)	0.229		
TNM stage	I	reference	<0.001	reference	0.001	reference	0.784		
	II	1.105 (0.804–1.518)		1.192 (0.882–1.611)		1.173 (0.624–2.206)			
	III	2.167 (1.104–4.255)		1.823 (0.961–3.455)		0.000(0.000-.)			
	IVa	2.088 (1.592–2.739)		1.657 (1.273–2.157)		0.766 (0.390–1.503)			
Cirrhotic nodule	no	reference	0.274	reference	0.799	reference	0.067		
	yes	1.127 (0.910–1.397)		1.028 (0.834–1.266)		0.614 (0.365–1.035)			
DD	I	reference	0.982	reference	0.997	reference	1.000		
	II-III	2997.401 (0.000-.)		8142.694 (0.000-.)		300,921,742.177 (0.000-.)			
	IV	2632.881 (0.000-.)		8450.077 (0.000-.)		1.000 (0.000-.)			
IBL	<1000 mL	reference	0.005	reference	0.002	reference	<0.001	reference	<0.001
	≥1000 mL	1.733 (1.184–2.535)		1.816 (1.243–2.652)		65.443 (22.383–191.341)		168.205 (43.249–654.184)	
Preventive TACE	no	reference	0.349	reference	0.144	reference	0.972		
	yes	0.841 (0.585–1.209)		1.300 (0.914–1.848)		1.015 (0.439–2.348)			

OS: overall survival; DFS: disease-free survival; OR: odd ratio; HR: hazard ratio; “.”: indicate the exceeded value

^a^likelihood ratio test

### Demographics and clinical characteristics of 215 matched patients after PSM

Before performing propensity score matching analysis, we used a calculation called “PS Power and Sample Size Calculations(Version 3.0, January 2009)” [21, 22] to estimate the ideal sample size. We are planning a study with 1 PBT subject matched to 4 no-PBT subjects, an accrual interval of 1 month, and additional follow-up after the accrual interval of 133 months. Prior data indicate that the median survival time on the no-PBT is 26 months. If the true median survival times on the PBT and no-PBT are 26 and 12 months, respectively, we will need to study 31 PBT subjects and 124 no-PBT subjects to be able to reject the null hypothesis that the experimental and control survival curves are equal with probability (power) 0.9(*β* = 0.1). The Type I error probability associated with this test of this null hypothesis is 0.01(*α*).

**Fig. 2:**
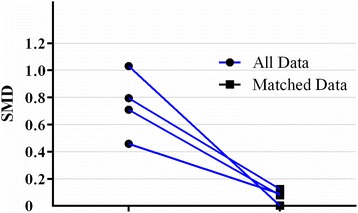
The model values of absolute standard mean difference(SMD) before and after PSM. The SMD of propensity score and three confounders (Preoperative Hb, Total bilirubin, intraoperative blood loss) was depicted in all data round dot. The SMD of matched data was depicted in squared dot

The propensity score matching procedure was performed to reduce confounding variables based on the three identified factors. The caliper was set at 0.05, and we used an optimal match ratio of 1:4. We found 52 of the 93 transfused patients were matched with 163 of the 512 no-transfused patients, which is more than the ideal sample size we calculated previously to obtain the significant conclusion. The propensity score suggests there were no biases in the matched groups (0.136 ± 0.117 vs. 0.193 ± 0.167, *P* = 0.785). Figure 2 shows the matched data absolute standardized mean difference (SMD), and the SMD of all three confounders and PS decreased to less than 0.2. In Table 3, the matched patient characteristics were compared, and no significant differences were shown between the groups, considering all 21 variables.

**Table 3:** Demographics and clinical characteristics of 215 patients after PSM

Characteristic	Variable	After PSM		
		No-PBT	PBT	*P* value
		(*n* = 163)	(*n* = 52)	
Age	Y ± SD	57.67 ± 10.77	56.90 ± 12.34	0.114^a^
Gender	female/male	72/91	27/25	0.330^c^
Preoperative Hb	g/L ± SD	125.38 ± 15.90	120.25 ± 18.72	0.232^a^
Platelet	1 × 10^9^/L ± SD	180.29 ± 65.50	193.38 ± 93.35	0.350^a^
Total bilirubin	umol/L ± SD	31.34 ± 62.77	60.36 ± 115.37	0.070^b^
AST	available data	161 (98.77%)	50 (96.15%)	
	U/L ± SD	46.25 ± 81.74	47.92 ± 62.24	0.763^b^
ALT	U/L ± SD	51.14 ± 80.64	53.35 ± 77.74	0.374^b^
AFP	available data	158 (96.93%)	52 (100.00%)	
	ng/mL ± SD	196.77 ± 1590.50	123.49 ± 741.51	0.994^b^
CEA	available data	157 (96.32%)	49 (94.23%)	
	ng/mL ± SD	22.66 ± 149.81	95.03 ± 437.78	0.375^b^
CA19–9	available data	156 (95.71%)	48 (92.31%)	
	U/mL ± SD	1170.62 ± 3041.71	1491.47 ± 3691.9	0.439^b^
PT	available data	161 (98.77%)	51 (98.08%)	
	s ± SD	10.76 ± 4.65	11.96 ± 3.59	0.075^b^
INR	available data	158 (96.93%)	50 (96.15%)	
	Value ±SD	0.98 ± 0.13	0.98 ± 0.15	0.997^b^
HBsAg	available data	160 (98.16%)	52 (100.00%)	
	(−)/(+)	100/60	39/13	0.100^c^
Anti-HCV	available data	159 (97.55%)	52 (100.00%)	
	(−)/(+)	157/2	51/1	0.726^c^
Tumor number	1/>1	140/23	43/9	0.574^c^
TMD	cm	6.84 ± 3.39	6.83 ± 3.38	0.967^c^
TNM stage	available data	155(95.09%)	44 (84.62%)	
	I/II/III/IVa	92/24/5/34	25/11/0/8	0.968^c^
Cirrhotic nodule	available data	163 (100%)	50 (96.2%)	
	no/yes	112/51	40/10	0.123^c^
DD	available data	126 (77.30%)	43 (82.70%)	
	I/ II-III	2/124 (1.59%)	0/43 (0.00%)	0.407^c^
IBL	<1 L/≥1 L	161/2 (98.77%)	52/0 (100.00%)	0.423^c^
Preventive TACE	no/yes	153/10 (93.87%)	47/5 (90.38%)	0.392^c^
Propensity Score		0.136 ± 0.117	0.193 ± 0.167	0.785^d^

^a^t test

^b^K-Independent-Samples Test (Kruskal Wallis H(K) test)

^c^Two-Independent-Samples Test (Mann-Whitney U test)

^d^Two-Related-Samples Test (Wilcoxon Signed Ranks Test)

Values are presented as n (%) or mean ± standard deviation

### Perioperative blood transfusion has no effect on OS and DFS after PSM

**Fig. 3:**
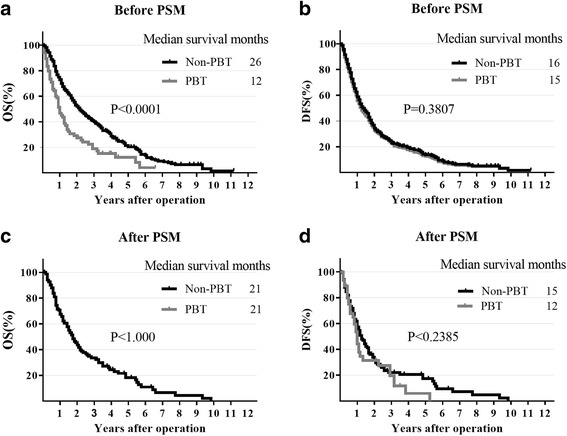
Kaplan-Meier survival plot of OS and DFS before and after PSM. The survival curve of overall survival and disease-free survival in unadjusted model (**a**, **b**). The survival curve of overall survival and disease-free survival after matched (**c**, **d**). Median survival months were showed in each figure

The univariate Cox proportional hazards regression analysis indicated PBT has a poor effect on OS and DFS before propensity score matching, which forebodes an 88.9% risk of overall mortality (HR: 1.889, 95% CI: 1.446–2.468, *p* < 0.001) and 58.9% risk of DFS (HR: 1.589, 95% CI: 1.221–2.067, *p* < 0.001). Additionally, the Kaplan-Meier curve of OS showed the no-transfused group has a significant benefit compared with transfused group (*p* < 0.0001) (Fig. 3), the median survival months(MSMs) of PBT (12 months) is obviously less than non-PBT (26 months), Of note, no difference was found in DFS (*p* = 0.3807), same as the MSMs (PBT = 15 months, non-PBT = 16 months). However, after performing a multivariate risk dependent Cox regression we found that neither OS (HR: 1.172, 95% CI: 0.756–1.816, *p* = 0.479) nor DFS (HR: 0.944, 95% CI: 0.608–1.466, *p* = 0.799) was significantly different due to blood transfusion. Our findings suggest it was not a statistically independent prognostic risk. After propensity score matching, PBT had no significant effect on the risk of OS (HR: 1.429, 95% CI: 0.972–2.103, *p* = 0.070) and DFS (HR: 1.262, 95% CI: 0.858–1.856, *p* = 0.238) (Table 4). Furthermore, the Kaplan-Meier plot showed similar trends for OS (*P* < 1.000) and DFS (*P* < 0.230). Both PBT and non-PBT MSMs to OS is 21 months and no significant difference (PBT = 12 months, non-PBT = 15 months) to DFS.

**Table 4:** Univariate and multivariate Cox analysis predicting OS and DFS based on Transfusion and No-transfusion

Characteristic	Variable	Before PSM				After PSM	
		univariate		multivariate		univariate	
		HR (95% CI)	*P* value^a^	HR (95% CI)	*P* value^a^	HR (95% CI)	*P* value^a^
PBT(OS)	no	reference	<0.001	reference	0.479	reference	0.070
	yes	1.889 (1.446–2.468)		1.172 (0.756–1.816)		1.429 (0.972–2.103)	
PBT(DFS)	no	reference	0.001	reference	0.799	reference	0.238
	yes	1.589 (1.221–2.067)		0.944 (0.608–1.466)		1.262 (0.858–1.856)	

^a^likelihood ratio test

## Discussion

Several studies have focused on how PBT affects gastrointestinal carcinomas and other tumors [6–8, 10, 11, 14, 23, 24], and some concluded that PBT led to a poor outcome and increased the probability of recurrence [6–8, 25, 26]]. However, others reported that PBT was not an independent prognostic factor for tumor recurrence and OS [24]. Müller et al. reached the same conclusion in a study of 128 advanced cholangiocarcinoma patients [9]: the small number of sources and heterogeneity from a mixture of intrahepatic, hilar, and distal cholangiocarcinoma may result an unconvincing selection bias. In our consecutive retrospective cohort study with 758 patients, we only recruited those who had intrahepatic cholangiocarcinoma without metastasis, and we found that PBT had no prognosis-related effect on OS and DFS.

We first explored the variables in 605 patients and found a significant bias between the two groups, which is consistent with previous reports. The survival analysis showed transfusion was an independent prognostic cause of OS and DFS. Moreover, we tried to determine which variables interacted with the outcome and treatment, using a combination of logistic and Cox regressions, and we found three interference factors: preoperative Hb, total bilirubin, and intraoperative blood loss. The propensity score matching was performed to reduce the confounding influence based on the three factors. To obtain the optimal matching data, the match ratio of 1:4 and a general caliper value of 0.05 were applied based on Abadie’s research [17]. We matched 52 transfused patients to 163 no-transfused patients, and all SMD values were less than 0.2, which suggests that the matching model was well adjusted [27]. A further exploration of all variables was performed using the same statistical method, and no significant bias persisted between the groups in terms of the *P* value and PS. The survival analysis of OS and DFS also revealed that PBT had no effect on OS or DFS. Admittedly, we could not deny its effect on delaying the duration of the hospital stay, a higher probability of complication such as febrile reaction, allergic reaction, graft-vs. -host disease (GVHD), hemolytic reaction, and other long-term results like virus infection and immunosuppression.

Fundamental confounding factors and inappropriate statistical methods may result in the illusion that PBT may lead to poor survival. Lian X et al. demonstrated the positive viewpoint by using a large gastric adenocarcinoma data set [6]. They revealed that PBT resulted in poor prognosis but not an independent prognostic factor based on a univariate and multivariate Cox analysis. The explanation might be different distribution of clinicopathological features between two groups and some confounder existed, such as TNM stage and intraoperative blood loss. Our ICC data set indeed found that intraoperative blood loss is an important confounder, in fact, it is more likely to transfuse blood for those massively bleeding patients. Norihisa Kimura et al. concluded that PBT was a strong risk factor for both recurrence and poor survival based on 66 HCCA after aggressive surgical resection [8], they also kindly pointed out their limit of the small sample size may have resulted in a loss of statistical power. Finding that such confounders could adversely cover up the truth. Therefore, an appropriate technique such as PSM must be performed to avoid the bias before the final analysis, and ideal sample size of matched pairs may be necessary to strength the conclusion.

There are several limitations in this study. First, although the propensity score matching analysis is an acceptable method of simulating a random controlled trial, but it is still not sufficient to make up for the value of RCTs in circumstances with ethical challenges. Second, our study only recruited patients within China, and the results may not be applicable to other countries especially western states. Third, the exclusion of patients in the propensity score matching analysis reduced the statistical power. Finally, unknown or unobserved confounding factors may contribute to potential bias because the missing source collection and available data may result in information bias.

## Conclusions

In conclusion, our data suggest that PBT was not associated with the long-term outcome of ICC. Inappropriate statistical analyses may lead to variable results, and risk adjustments can eliminate the detrimental effect.

## Abbreviations

AFP: alpha fetoprotein
ALT: alanine transaminase
Anti-HCV: anti-hepatitis C virus
AST: aspartate aminotransferase
CA19–9: carbohydrate antigen 19–9
CEA: carcinoembryonic antigen
DD: The degree of differentiation
DFS: disease-free survival
Hb: hemoglobin
HBsAg: hepatitis B surface antigen
HR: hazard ratio
IBL: intraoperative blood loss
ICC: intrahepatic cholangiocarcinoma
INR: international normalized ratio
MSMs: median survival months
No-PBT: no-perioperative transfusion
OR: odd ratio
OS: overall survival
PBT: perioperative blood transfusion
PBT: perioperative transfusion
PS: propensity score
PSM: propensity score matching
PT: prothrombin time
RCT: random controlled trials
SMD: absolute standardized mean difference
TACE: transcatheter arterial chemoembolization
TMD: tumor maximum dimension
TNM: tumor node metastasis

## References

[1] Torre LA, Sauer AMG, Chen MS, Kagawa-Singer M, Jemal A, Siegel RL (2016). Cancer statistics for Asian Americans, native Hawaiians, and Pacific islanders, 2016: converging incidence in males and females. CA Cancer J Clin.

[2] Dodson RM, Weiss MJ, Cosgrove D, Herman JM, Kamel I, Anders R (2013). Intrahepatic cholangiocarcinoma: management options and emerging therapies. J Am Coll Surgeons.

[3] Chen W, Zheng R, Baade PD, Zhang S, Zeng H, Bray F (2016). Cancer statistics in China, 2015. CA Cancer J Clin.

[4] Siegel RL, Miller KD, Jemal A (2016). Cancer statistics, 2016. CA Cancer J Clin.

[5] Chen W, Zheng R, Zhang S (2013). Liver cancer incidence and mortality in China, 2009. Chinese J Cancer.

[6] Xue L, Chen X, Wei-Han Z, Yang K, Chen X, Zhang B, et al. Impact of perioperative blood transfusion on postoperative complications and prognosis of gastric adenocarcinoma patients with different preoperative hemoglobin value. Gastroent Res Pract. 2016;(1):1–10. 10.1155/2016/6470857 PMC4706942 26819609

[7] Mavros MN, Xu L, Maqsood H, Gani F, Ejaz A, Spolverato G (2015). Perioperative blood transfusion and the prognosis of pancreatic cancer surgery: systematic review and meta-analysis. Ann Surg Oncol.

[8] Kimura N, Toyoki Y, Ishido K, Kudo D, Yakoshi Y, Tsutsumi S (2015). Perioperative blood transfusion as a poor prognostic factor after aggressive surgical resection for hilar cholangiocarcinoma. J Gastrointest Surg.

[9] Müller SA, Mehrabi A, Rahbari NN, Warschkow R, Elbers H, Leowardi C (2014). Allogeneic blood transfusion does not affect outcome after curative resection for advanced cholangiocarcinoma. Ann Surg Oncol.

[10] De Boer MT, Molenaar IQ, Porte RJ: Impact of blood loss on outcome after liver resection. Digest Surg 2007, 24(4):259–264. 10.1159/000103656 17657150

[11] Warschkow R, Güller U, Köberle D, Müller SA, Steffen T, Thurnheer M (2014). Perioperative blood transfusions do not impact overall and disease-free survival after curative rectal cancer resection. Ann Surg.

[12] Yang T, Lu J, Lau WY, Zhang T, Zhang H, Shen Y (2016). Perioperative blood transfusion does not influence recurrence-free and overall survivals after curative resection for hepatocellular carcinoma a propensity score matching analysis. J Hepatol.

[13] Rizvi S, Gores GJ. Pathogenesis, Diagnosis, and Management of Cholangiocarcinoma 2013 Gastroenterology. Gastroenterol. 2013(145):1215–29. 10.1053/j.gastro.2013.10.013 PMC3862291 24140396

[14] Salpeter SR, Buckley JS, Chatterjee S (2014). Impact of more restrictive blood transfusion strategies on clinical outcomes: a meta-analysis and systematic review. Am J Med.

[15] Abadie A, Angrist J, Guido I (2002). Instrumental variable estimates of the effect of subsidized training on the quantile of trainee earnings. Econometrica.

[16] Mirici-Cappa F, Gramenzi A, Santi V, Zambruni A, Di Micoli A, Frigerio M (2010). Treatments for hepatocellular carcinoma in elderly patients are as effective as in younger patients: a 20-year multicentre experience. Gut.

[17] Abadie A, Imbens GW (2011). Bias-corrected matching estimators for average treatment effects. J Bus Econ Stat.

[18] Williamson EJ, Forbes A (2014). Introduction to propensity scores. Respirology.

[19] Kim DH, Pieper CF, Ahmed A, Colón-Emeric CS (2016). Use and interpretation of propensity scores in aging research: a guide for clinical researchers. J Am Geriatr Soc.

[20] The R Project for Statistical Computing. https://www.r-project.org/. Accessed 1 May 2016.

[21] Dupont WD, Plummer WD (1990). Power and sample size calculations. Control Clin Trials.

[22] Dupont WD, Plummer WD (1998). Power and sample size calculations for studies involving linear regression. Control Clin Trials.

[23] Jarnagin WR (2002). Improvement in perioperative outcome after hepatic resection. Ann Surg.

[24] Han S, Kim G, Ko JS, Sinn DH, Yang JD, Joh J (2016). Safety of the use of blood salvage and autotransfusion during liver transplantation for hepatocellular carcinoma. Ann Surg.

[25] Acheson AG, Brookes MJ, Spahn DR (2012). Effects of allogeneic red blood cell transfusions on clinical outcomes in patients undergoing colorectal cancer surgery: a systematic review and meta-analysis. Ann Surg.

[26] Wada H, Eguchi H, Nagano H, Kubo S, Nakai T, Kaibori M, et al. Perioperative allogenic blood transfusion is a poor prognostic factor after hepatocellular surgery: a multi-center analysis. Surg Today. 2017; 10.1007/s00595-017-1553-3 28597349

[27] Baek S (2015). Propensity score matching: a conceptual review for radiology researchers. Korean Soc Radiol.

